# Distinct Geographical Distribution of the *Miscanthus* Accessions with Varied Biomass Enzymatic Saccharification

**DOI:** 10.1371/journal.pone.0160026

**Published:** 2016-08-17

**Authors:** Xukai Li, Haofeng Liao, Chunfen Fan, Huizhen Hu, Ying Li, Jing Li, Zili Yi, Xiwen Cai, Liangcai Peng, Yuanyuan Tu

**Affiliations:** 1 Biomass and Bioenergy Research Centre, Huazhong Agricultural University, Wuhan, China; 2 National Key Laboratory of Crop Genetic Improvement, Huazhong Agricultural University, Wuhan, China; 3 College of Plant Science and Technology, Huazhong Agricultural University, Wuhan, China; 4 Department of Biotechnology, Hunan Agricultural University, Changsha, China; 5 Department of Plant Science, North Dakota State University, Fargo, North Dakota, United States of America; University of Huddersfield, UNITED KINGDOM

## Abstract

*Miscanthus* is a leading bioenergy candidate for biofuels, and it thus becomes essential to characterize the desire natural *Miscanthus* germplasm accessions with high biomass saccharification. In this study, total 171 natural *Miscanthus* accessions were geographically mapped using public database. According to the equation [P(H/L| East) = P(H/L∩East)/P(East)], the probability (P) parameters were calculated on relationships between geographical distributions of *Miscanthus* accessions in the East of China, and related factors with high(H) or low(L) values including biomass saccahrification under 1% NaOH and 1% H_2_SO_4_ pretreatments, lignocellulose features and climate conditions. Based on the maximum P value, a golden cutting line was generated from 42°25’ N, 108°22’ E to 22°58’ N, 116°28’ E on the original locations of *Miscanthus* accessions with high P(H|East) values (0.800–0.813), indicating that more than 90% *Miscanthus* accessions were originally located in the East with high biomass saccharification. Furthermore, the averaged insolation showed high P (H|East) and P(East|H) values at 0.782 and 0.754, whereas other climate factors had low P(East|H) values, suggesting that the averaged insolation is unique factor on *Miscanthus* distributions for biomass saccharification. In terms of cell wall compositions and wall polymer features, both hemicelluloses level and cellulose crystallinity (CrI) of *Miscanthus* accessions exhibited relative high P values, suggesting that they should be the major factors accounting for geographic distributions of *Miscanthus* accessions with high biomass digestibility.

## Introduction

*Miscanthus*, a typical C4 perennial grass with extremely high biomass yield, is currently considered a leading bioenergy crop for the production of biofuel feedstock [1–4]. *Miscanthus* has been historically utilized in the industries of forage, textile, and shelter. As a fast-growing grass, it requires little water and fertilization, and can thus grow in poor soil with minimal inputs [5, 6]. *Miscanthus* is a genus of about 20 species and distributes widely under various climate conditions across Asia and the nearby Pacific islands [5]. In China, at least seven species have been found, and each species has a unique geographical distribution [7–10]. Because of its self-incompatible property, *Miscanthus* has a diverse genetic variation. Recent reports indicated that Chinese *Miscanthus* accessions exhibited diverse cell wall compositions and wall polymer features for largely varied biomass saccharification [10, 11].

Plant cell walls represent a large biomass resource for biofuel and other chemical products. However, lignocellulose recalcitrance greatly affects cost-effectiveness of biomass processing that involves in three major steps: physical and chemical pretreatment, enzymatic hydrolysis, and yeast fermentation [12, 13]. Recalcitrance is determined mainly by the cell wall composition and wall polymer features [14–17]. Genetic improvement of energy crops is proposed as a promising solution for reducing recalcitrance [10, 17, 18].

Cellulose is a major cell wall polysaccharide that accounts for 20% to 35% of the dry matter of grasses, and its crystallinity is reportedly a negative factor affecting biomass hydrolysis in plants [14, 19, 20]. Xylan is a major hemicellulose that positively affects biomass enzymatic saccharification by reducing lignocellulose crystallinity (CrI) in *Miscanthus* [15, 16]. Furthermore, the arabinose (Ara) substitution degree (reverse Xylose/Ara) of xylan could enhance biomass saccharification under various chemical pretreatments in *Miscanthus* [16]. Lignin is an amorphous heteropolymer containing three major phenolic components: *p*-coumaryl alcohol (H), coniferyl alcohol (G), and sinapyl alcohol (S) [21, 22]. Recent reports have indicated that lignin is a negative factor on biomass digestibility under various physical and chemistry pretreatments in *Miscanthus*, the S/G ratio determines the negative effect of lignin on biomass digestibility in *Miscanthus* [15, 23, 24].

In this work, we selected 171 *Miscanthus* accessions that have been reported to represent four major species from a large natural *Miscanthus* collection (n = 1400) around China [10, 11], and mapped the geographical distribution of each species. Also, we performed a correlation analysis of cell wall composition/wall polymer features, biomass saccharification, and meteorological factors at the geographical location of each species. Furthermore, we characterized and selected the *Miscanthus* accessions for high biomass enzymatic digestion. These informations will be useful for*Miscanthus* breeding toward the development of desirable bioenergy crop.

## Materials and Methods

### Materials

**Fig 1:**
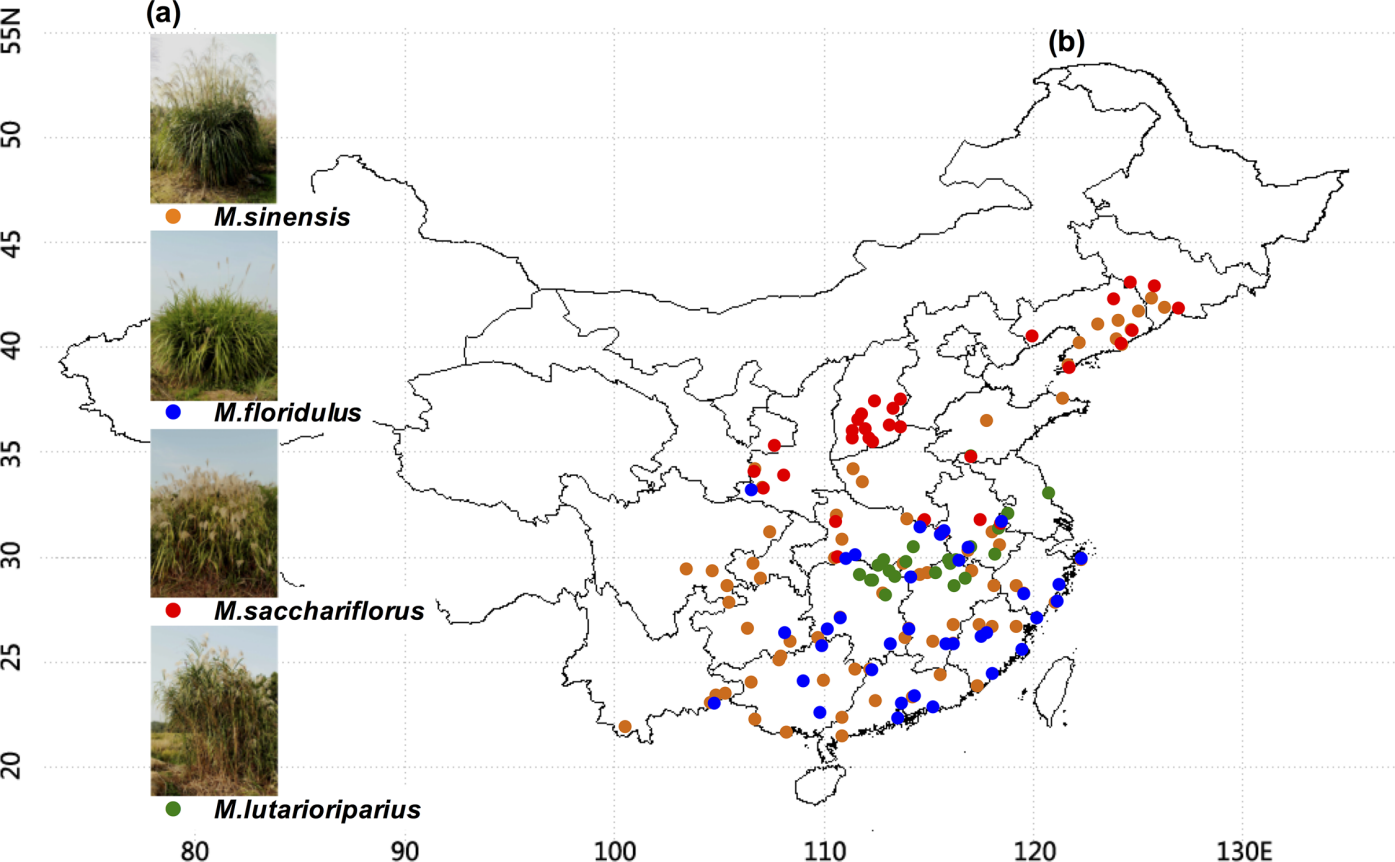
Geographic distribution of four *Miscanthus* species in China. (**a**) *Miscanthus* growth; (**b**) Map of *Miscanthus* locations.

A total of 171 natural *Miscanthus* accessions under four major species (*M*.*sinensis*, *M*. *floridulua*, *M*. *saccharifloru*s, and *M*. *lutarioriparius*) were collected in China in 2007 (Table 1, Fig 1 and S1 File). The samples harvested from Hunan experimental field in the 2009 and 2010 seasons were dried at 50°C after treated at 105°C for 5 min. The dried tissues were ground through a 40 meshes screen and stored in a dry container until use.

**Table 1:** Variation of meteorological factors in four major *Miscanthus* species.

Species	Number of Accessions	Longitude (E) Latitude (N)	Average Temperature (°C)	CV% ^a^	Lowest Temperature (°C)	CV%	Average Insolation (kWh/m^2^/day)	CV%	Average Precipitation (mm/day)	CV%
*M*.*sinensis*	78	100.59~126.40	16.13*±4.33	26.85	11.46±5.29	46.15	3.67±0.40	10.99	3.48±0.95	27.26
		18.81~42.38	(4.34~25.70) ^**#**^		(-2.27~23.87)		(2.88~4.67)		(1.65~5.00)	
*M*.*floridulus*	36	104.79~122.39	18.44±2.91	15.76	14.17±3.95	27.87	3.61±0.30	8.39	4.09±0.65	16.00
		19.14~33.16	(9.93~24.60)		(4.46~22.19)		(3.14~4.52)		(1.99~5.30)	
*M*.*sacchariflorus*	32	106.75~126.98	11.84±3.18	26.89	5.67±3.65	64.34	4.05±0.31	7.58	2.05±0.71	34.73
		30.03~43.08	(4.34~17.30)		(-2.27~11.90)		(3.34~4.46)		(1.36~3.54)	
*M*.*lutarioriparius*	25	111.77~120.81	17.06±0.61	3.55	11.73±0.69	5.86	3.55±0.23	6.42	3.70±0.47	12.67
		28.18~34.82	(15.70~17.80)		(9.92~12.68)		(3.24~4.11)		(2.24~4.44)	
Total	171	100.59~126.98	15.95±4.10	25.72	10.99±5.10	46.44	3.71±0.38	10.26	3.37±1.04	30.76
		18.81~43.08	(4.34~25.70)		(-2.27~23.87)		(2.88~4.67)		(1.36~5.30)	

* Mean value

^**#**^ range.

^a^ The coefficient of variation (CV), which represents the ratio of the standard deviation to the mean, is a statistical measure of the dispersion of data points in a data series around the mean.

### Wall polysaccharide extraction

The plant cell wall fractionation method was used for extraction of cellulose and hemicelluloses as described by Peng LC *et al*. [25] and Xu N *et al*. [15]. The crude cell wall material was suspended in 0.5% (w/v) ammonium oxalate and heated for 1 h in a boiling water bath, and the supernatants were combined as total pectin. The remaining pellet was suspended in 4 M KOH containing 1.0 mg /mL sodium borohydride for 1 h at 25°C, and the combined supernatant was neutralized, dialyzed and lyophilized as hemicelluloses. The KOH non-extractable residue was further extracted with acetic-nitric acids for 1 h at 100°C and the remaining pellet was defined as crystalline cellulose. All experiments were carried out in biological triplicate.

### Colorimetric assay for hexoses and pentoses

UV/VIS Spectrometer (Shanghai MAPADA Instruments Co., Ltd. V-1100D) was used for total hexose and pentose measurement. Hexose content was determined by the anthrone (Sigma-Aldrich Co. LLC) /H_2_SO_4_ method [26]. Cellulose was dissolved in 67% (v/v) H_2_SO_4_ (1.0 mL) with shaking at 25°C for 1 h, and then 10.0 0μL aliquot was used to determine cellulose content and hexoses yield released from pretreatment and enzymatic hydrolysis using the anthrone/H_2_SO_4_ method. Pentose content was measured by the orcinol/HCl method [26], and the assay was used to determine pentose yield released from pretreatment and enzymatic hydrolysis. Because the high pentose level in the sample can affect the absorbance reading at 620 nm for hexose content by anthrone/H_2_SO_4_ method, the deduction from pentose reading at 660 nm was carried out for final calculation of hexose level. All experiments were carried out in triplicate.

### Detections of hemicellulosic monosaccharides and cellulose crystallinity

Trifluoroacetic acid (TFA) and *myo*-inositol were purchased from Aladdin Reagent Inc. Acetic anhydride and acetic acid were obtained from Sinopharm Chemical Reagent Co., Ltd. 1-Methylimidazole was purchased from Sigma–Aldrich Co. LLC. Monosaccharide standards including l-rhamnose, l-arabinose, l-fucose, d-xylose, d-galactose, d-glucose and d-mannose, were obtained from Sinopham Chemical Reagent Co., Ltd. The combined supernatants from 4 M KOH fraction were dialyzed for 36 h after neutralization with acetic acid. The polysaccharides dissolved in 2.5 mL TFA (2 M) were heated in a sealed tube at 121°C in an autoclave (15 psi) for 1 h. *Myo*-inositol (200 μg) was added as the internal standard. The supernatant was dried under vacuum at 38°C to remove TFA. Distilled water (800 μL) and a freshly prepared solution of NaBH_4_ (400 μL, 100 mg/mL in 6.5 M aqueous NH_3_) were added to each sample. Sample was capped, mixed well and incubated at 40°C for 30 min. Excess NaBH_4_ was decomposed by adding acetic acid (800 μL). 400 μL Sample was then moved into a 25 mL glass tube. Acetic anhydride (4 mL) was added to the tube and the solution mixed again. Then 1-methylimidazole (600 μL) was added. After mixing, the sample was allowed to stand for 10 min. Excess acetic anhydride was decomposed by adding distilled water (10 mL). Then dichloromethane (3 ml) was added, mixed gently, centrifuged (2,000 g, 10 seconds) for phase separation. After removing the upper phase, the sample was washed with distilled water (3 × 20.0 mL). The collected lower phase was dehydrated by adding with anhydrous sodium sulfate and stored at −20°C until analyzed by GC-MS. The sample preparation and GC–MS (SHIMADZU GCMS-QP2010 Plus) running were previously described by Li FC *et al*. [16]. Restek Rxi-5 ms, 30 m × 0.25 mm ID × 0.25um df column. Carrier gas: He. Injection Method: Split. Injection port: 250°C, Interface: 250°C. Injection Volume: 1.0 μL. The temperature program: from 170°C (held for12 min) to 220°C (held for 8 min) at 3°C/min. Ion source temperature: 200°C, ACQ Mode: SIM. The mass spectrometer was operated in the EI mode with ionization energy of 70 ev. Mass spectra were acquired with full scans based on the temperature program from 50 to 500 m/z in 0.45 s. Calibration curves of all analytes routinely yielded correlation coefficients 0.999 or better.

X-ray diffraction (XRD) method was used to determine cellulose crystallinity index (CrI) using Rigaku-D/MAX instrument (Uitima III, Japan) as described by Zhang W *et al*. [14]. The raw biomass powder was laid on the glass sample holder (35 × 50 × 5 mm) and detected under plateau conditions. Ni-filtered Cu Kα radiation (λ = 0.154056 nm) generated at voltage of 40 kV and current of 18 mA, and scanned at speed of 0.0197°/s from 10–45°. The crystallinity index (CrI) was estimated using the intensity of the 200 peak (I_200_, θ = 22.5°) and the intensity at the minimum between the 200 and 110 peaks (I_am_, θ = 18.5°) as the follow: CrI = 100 × (I_200_−I_am_)/I_200_. I_200_ represents both crystalline and amorphous materials while I_am_ represents amorphous material. Standard error of the CrI method was determined at ±0.05~0.15 using five representative samples in triplicate.

### Total lignin and monolignol assay

Total lignin was determined by the two-step acid hydrolysis method according to Laboratory Analytical Procedure of the National Renewable Energy Laboratory. The lignin includes acid-insoluble and -soluble lignin. The acid-insoluble lignin was calculated gravimetrically after correction for ash, and the acid-soluble lignin was measured by UV spectroscopy. For acid-insoluble lignin determination, a 0.5 g sample recorded as W1. Each sample was run in triplicate. The sample was extracted with benzene-ethanol (2:1, v/v) in a Soxhlet for 4 h, and then air-dried in hood overnight. The sample was hydrolyzed with 10 mL 72% H_2_SO_4_ (v/v) in shaker at 30°C for 1.5 h. After hydrolysis, the acid was diluted to a concentration of 2.88%, and then placed in the autoclave for 1 h at 121°C (15 psi). The autoclaved hydrolysis solution was vacuum-filtered through the previously weighed filtering crucible. The filtrate was captured in a filtering flask for acid-soluble lignin. The lignin was washed free of acid with hot distilled water and the crucible and acid-insoluble residue was dried in an oven at 80°C until constant weight was achieved. Then, the samples were removed from the oven and cool in a dry-container. The weight of the crucible and dry residue was recorded to the nearest 0.1 mg (W2). The dried residue was ashed in the muffle furnace at 200°C for 30 min and 575°C for 4 h. The crucibles and ash were weighed to the nearest 0.1 mg and recorded the weight (W3). Acid-insoluble lignin (AIL) on original sample was calculated as the following: AIL (%)  =  (W2-W3) × 100/W1. For acid-soluble lignin determination, it was solubilized during the hydrolysis process, and was measured by UV spectroscopy. The hydrolysis liquor obtained previously was transfer into 250 mL volumetric flask and brought up to 250 mL with 2.88% sulfuric acid. The absorbance of the sample was read at 205 nm on a UV–vis spectroscopy (Beckman Coulter Inc., Du800), and 2.88% sulfuric acid was used as blank. The method of calculation about the amount of acid soluble lignin was as follows: ASL (%)  =  (A × D × V/1000 × K × W1) × 100%. A (absorption value), D (Dilution ratio of the sample), K (absorptivity constant)  =  110 L/g/cm. Total lignin (%)  =  ASL% + AIL%. All experiments were carried out in triplicate.

Three monolignols were measured using HPLC as described by Xu N *et al*. [10]. The standard chemicals, p-Hydroxybenzaldehyde (H), vanillin (G) and syringaldehyde (S) were purchased from Sinopharm Chemical Reagent Co., Ltd. The sample was extracted with benzene-ethanol (2:1, v/v) in a Soxhlet for 4 h, and the remaining pellet was collected as cell-wall residue (CWR). The procedure for nitrobenzene oxidation of lignin was conducted as follows: 0.05 g CWR was added with 5 mL 2 M NaOH and 0.5 mL nitrobenzene, and a stir bar was put into a 25-mL Teflon gasket in a stainless steel bomb. The bomb was sealed tightly and heated at 170°C (oil bath) for 3.5 h and stirred at 20 rpm. Then, the bomb was cooled with cold water. The chromatographic internal standard (ethyl vanillin) was added to the oxidation mixture. This alkaline oxidation mixture was washed three times with 30 mL CH_2_C1_2_/ethyl acetate mixture (1:1, v/v) to remove nitrobenzene and its reduction by-products. The alkaline solution was acidified to pH 3.0 to 4.0 with 6 M HCl, and then extracted with CH_2_C1_2_/ethyl acetate (3 × 30 mL) to obtain the lignin oxidation products, which were in the organic phase. The organic extracts were evaporated to dryness under reduced pressure at 40°C. The oxidation products were dissolved in 10 mL chromatographic pure methanol. For HPLC analysis the solution was filtered with a membrane filter (0.22 μm). Then, 20 μL solution was injected into the HPLC (Waters 1525 HPLC) column Kromat Universil C18 (4.6 mm × 250 mm, 5 μm) operating at 28°C with CH_3_OH:H_2_O:HAc (25:74:1, v/v/v) carrier liquid (flow rate: 1.1 mL/minute). Calibration curves of all analytes routinely yielded correlation coefficients 0.999 or higher, and the detection of the compounds was carried out with a UV-detector at 280 nm.

### Biomass pretreatment and enzymatic hydrolysis

Biomass pretreatments with NaOH and H_2_SO_4_ and sequential enzymatic hydrolysis were performed as described by Huang JF *et al*. [11] and Xu N *et al*. [15]. For NaOH pretreatment, the well-mixed powder of the biomass sample (0.5 g) was added with 10 mL NaOH at 1% (w/v). The tube was shaken at 150 rpm for 2 h at 50°C, and centrifuged at 3,000 *g* for 5 minutes. The pellet was washed three times with 10 mL distilled water, and stored at −20°C for enzymatic hydrolysis. All supernatants were collected for determination of total sugars (pentoses and hexoses) released from alkali pretreatment, and samples with 10 mL distilled water were shaken for 2 h at 50°C as the control. All samples were carried out in biological triplicate. For H_2_SO_4_ pretreatment, the well-mixed powder of the biomass sample (0.5 g) was added with 10 mL H_2_SO_4_ at 1% (v/v). The tube was sealed and heated at 121°C for 20 minutes in an autoclave (15 psi) after mixing well. Then, the tube was shaken at 150 rpm for 2 h at 50°C, and centrifuged at 3,000 g for 5 minutes. The pellet was washed three times with 10 mL distilled water, and stored at −20°C for enzymatic hydrolysis. All supernatants were collected for determination of total sugars released from acid pretreatment, and samples with 10 mL distilled water were shaken for 2 h at 50°C as the control. All samples were carried out in biological triplicate.

The remaining residues from various pretreatments were washed 2 times with 10 mL distilled water, and once with 10 mL mixed-cellulases reaction buffer (0.2 M acetic acid-sodium acetate, pH 4.8). The washed residues were added with 10 mL(2 g/L) mixed-cellulases (containing β-glucanase ≥ 6 × 10^4^ U) and cellulase ≥ 600 U and xylanase ≥ 10 × 10^4^ U from Imperial Jade Bio-technology Co., Ltd). During the enzymatic hydrolysis, the samples were shaking under 150 rpm at 50°C for 48 h. After centrifugation at 3,000 *g* for 10 min, the supernatants were collected for determining amounts of pentoses and hexoses released from enzymatic hydrolysis. The samples with 10 mL reaction buffer were shaken for 48 h at 50°C as the control. All samples were carried out in triplicate.

### Acquisition and analysis of climate data

The annual data for average and minimum earth's surface temperature, solar radiation, and rainfall for 171 locations in China were obtained from the National Aeronautics and Space Administration (NASA) Surface Meteorology and Solar Energy (SSE 6.0) website [27] (https://eosweb.larc.nasa.gov/sse/). The meteorological factors include average and minimum temperatures (°C), rainfall data over the 22-year period from Jan 1983 to Dec 2004; and solar radiation from July 1983 to Jun 2005.

### Draw the geographical distribution of the *Miscanthus* on map of China

The geographical distribution of the *Miscanthus* accessions were drawn in statistical computing and graphics language R (https://www.r-project.org/) using “maps” and “mapdata” packages.

### Determination of the golden cutting line and Bayes probabilities

**Fig 2:**
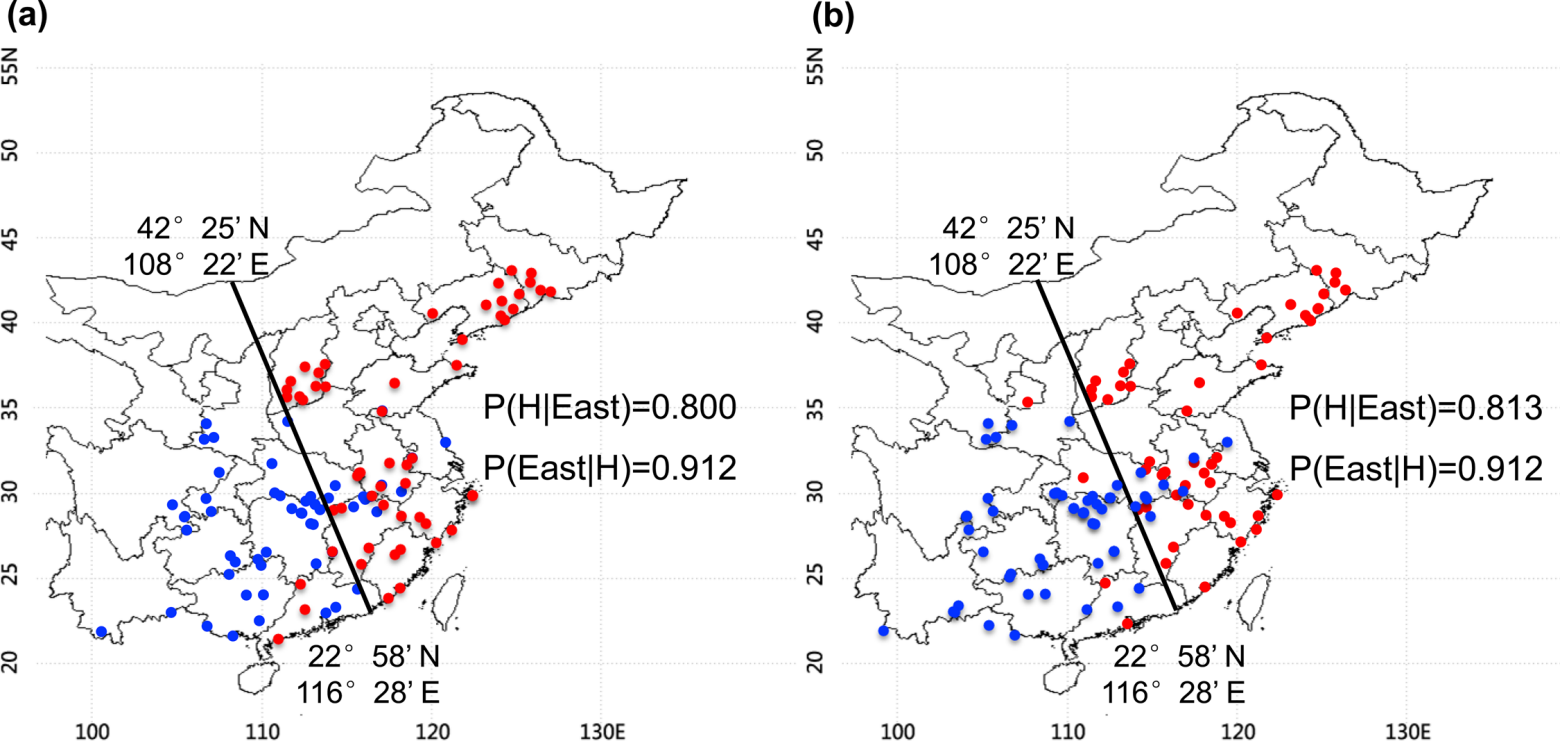
The cutting line of the geographic locations of 114 *Miscanthus* accessions with relatively high (H, dark dot) and low (L, gray dot) biomass saccharification under 1% NaOH (a) or 1% H_2_SO_4_ (b) pretreatment.

**Fig 3:**
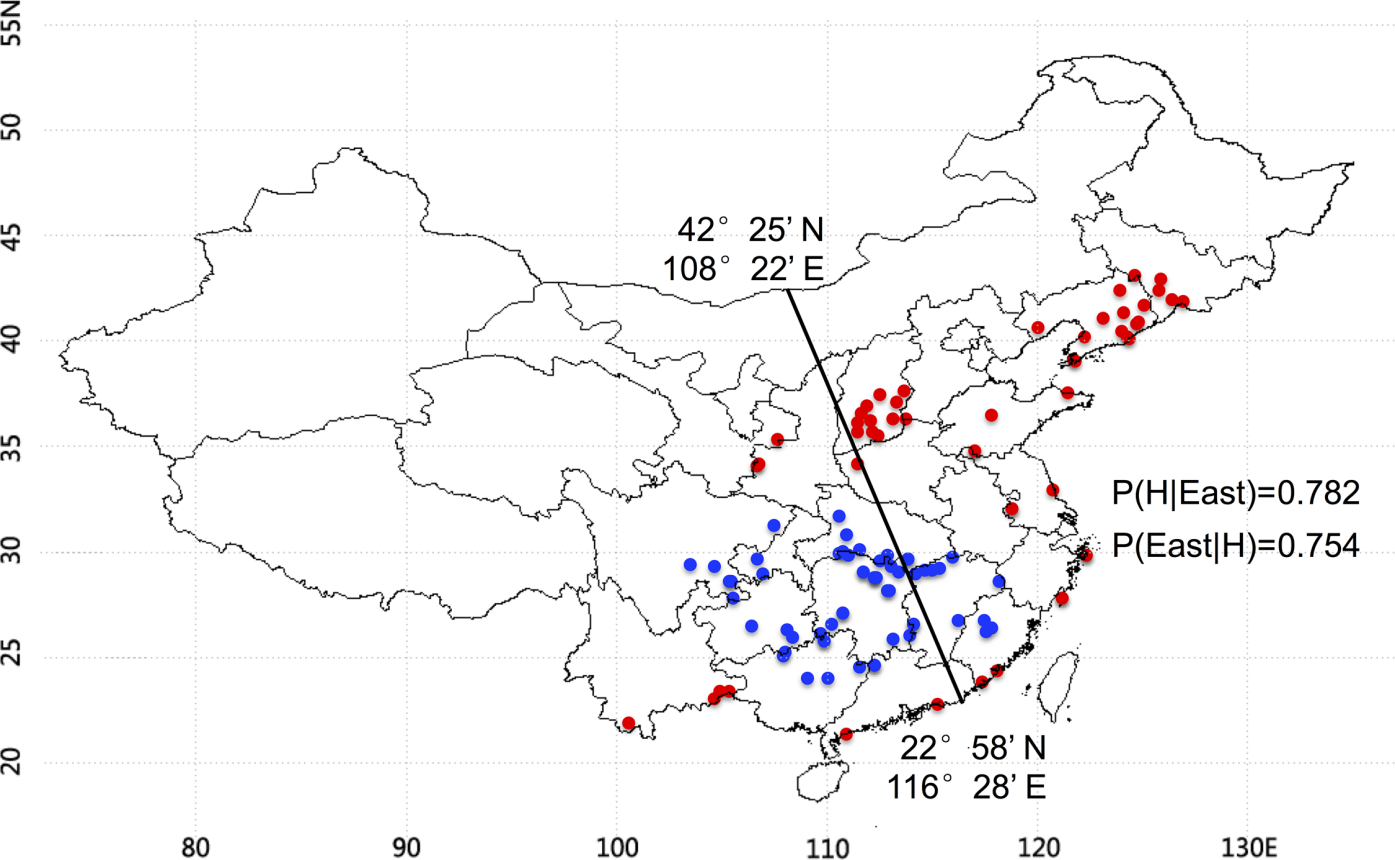
The cutting line of the geographic locations of 114 *Miscanthus* accessions under high (H, dark dot) and low (L, gray dot) average insolation (kWh/m2) in China.

Based on the biomass enzymatic digestibility under pretreatment with 1% NaOH or 1% H_2_SO_4_, the 171 *Miscanthus* accessions were equally divided into three groups with each containing 57 accessions. We mapped the geographic locations of Group I and Group III accessions, which had relatively high (H) and low (L) biomass digestibility, respectively. The boundary of high and low biomass digestibility samples on Chinese map are calculated using the Bayes theorem, and the method is known as the (empirical) Bayes method. It is a proper and standard statistical method. Suppose that the boundary divide China into two parts. Each part contains high and low biomass digestibility samples. To ensure the P(High | Area), a conditional probability of High given Area, is the maximum value by calculating the P parameter based on the equation: P(High | Area) = P(High∩Area) / P(Area). Then we got the cutting line (42°25’ N, 108°22’ E; 22°58’ N, 116°28’ E): Y = (301.46±6.26)-(2.395±0.055) X; (X means the Longitude (E) and Y means the Latitude (N)), and the line divide China into two parts: East and West areas (Figs 2 and 3). By this time, P(H | E) = P(High | Area).

We calculated the posterior probabilities P(E | H) using the Bayes' theorem (alternatively Bayes' law or Bayes' rule) based on the equation: P(E | H) = P(H) P(H | E) / P(E). This equation was also applied to measure the P parameter on the geographic locations of the samples based on their cell wall compositions and wall polymer features. In the equation: “E” indicates the *Miscanthus* accessions originally grown in the Eastern region of China based on the cutting lines; “H” indicates the *Miscanthus* samples with high biomass digestibility, or high wall polymer levels (cellulose, hemicelluloses, lignin) or high wall polymer features (CrI, Xyl/Ara, S/G).

## Results and Discussion

### Distinct geographic locations of four *Miscanthu*s species

A total of 171 representative *Miscanthus* accessions of four major species were selected from the collection of over 1400 natural *Miscanthus* accessions, including 78 *M*. *sinensis*, 36 *M*. *floridulus*, 32 *M*. *saccharifloru*s, and 25 *M*.*lutarioriparius* (Fig 1A). In general, the *Miscanthus* accessions were originally distributed from the longitude of 100.59 to 126.98 and latitude of 18.81 to 43.08 in China (Fig 1B, Table 1 and S1 File). However, each species exhibited a typical geographic location. *M*. *saccharifloru*s mainly distributed in the north, whereas almost all *M*. *floridulus* accessions were located in the south. In addition, *M*. *sinensis* had a broad distribution and *M*. *lutarioriparius* showed a narrow location. Due to the distinct geographic location, the *Miscanthus* accessions of each species were originally grown at typical climate conditions including average temperature, minimum temperature, average insolation, and average precipitation (Table 1). For instance, *M*. *floridulus* mainly grew under a relatively high average temperature (18.44 °C) and precipitation (4.09 mm/day), whereas most of the *M*. *sacchariflorus* accessions exposed to high average insolation (4.05 kWh/m2/day) and low average precipitation (2.05 mm/day). By comparison, *M*. *sinensis* exhibited relatively large variation in average and minimum temperatures (CV%: 26.85%, 46.15%), while *M*. *lutarioriparius* had small variation (3.55%, 5.86%). Hence, the four major *Miscanthus* species had distinct geographic distributions under typical climate conditions, suggesting that each *Miscanthus* species should grow under its typical ecosystem.

### Diverse cell wall compositions of four *Miscanthus* species

The *Miscanthus* accessions of the four species exhibited different cell wall compositions in addition to distinct geographic locations (Fig 1A, Table 2 and S1 File). For instance, *M*. *lutarioriparius* contained the highest levels of cellulose (45.89% of dry matter) and lignin (31.1%), whereas *M*. *sacchariflorus* had the lowest levels of cellulose (20.08%) and hemicellulose (23.01%). In addition, *M*. *floridulus* and *M*. *sinensis* exhibited the lowest lignin (19.28%) and the highest hemicelluloses (35.66%), respectively. Nevertheless, the four *Miscanthus* species were similar in the average hemicellulose levels ranging from 28.65% to 30.99% of the dry matter. A greater variation than hemicellulose was observed in the average cellulose contents ranging from 29.56% to 37.82%. These four *Miscanthus* species exhibited an intermediate variation of average lignin levels ranged from 22.94% to 27.73%. Furthermore, we examined cell wall compositions of 49 representative *Miscanthus* samples harvested in the 2009 and 2010 seasons, and found a significantly positive correlation of three wall polymers between two seasons at *p* < 0.01 level (Additional file 1), indicating that the characteristic cell wall compositions of the *Miscanthus* accessions did not respond differently to the growing seasons. In other words, the environmental conditions could not lead to much alteration of cell wall compositions and features in *Miscanthus* species.

**Table 2:** Variation of Cell wall compositions in four *Miscanthus* species.

Species	No. Accessions	Cell wall composition (% Dry matter)					
		Cellulose	CV%^a^	Hemicelluloses	CV%	Lignin	CV%
*M*.*sinensis*	78	31.60*	10.49	29.98	8.88	24.85	6.70
		(23.69~38.59) ^**#**^		(24.39~35.66)		(21.53~28.91)	
*M*.*floridulus*	36	30.06	12.47	28.65	7.59	22.94	8.12
		(22.27~37.52)		(25.01~33.94)		(19.28~26.31)	
*M*.*sacchariflorus*	32	29.56	19.20	30.38	8.03	27.14	5.14
		(20.08~44.15)		(23.01~34.80)		(23.55~29.95)	
*M*.*lutarioriparius*	25	37.82	13.80	30.99	7.15	27.73	5.91
		(28.47~45.89)		(26.61~34.56)		(25.10~31.10)	
Total	171	31.80	15.57	29.93	8.53	25.30	9.25
		(20.08~45.89)		(23.01~35.66)		(19.28~31.10)	

* Mean value

^**#**^ Ranges in parenthesis.

^a^ The coefficient of variation (CV),which represents the ratio of the standard deviation to the mean, is a statistical measure of the dispersion of data points in a data series around the mean.

### Biomass digestibility in the four *Miscanthus* species

Biomass digestibility (saccharification) was measured by either the hexose yield (hexoses/cellulose) released from the hydrolysis by a crude cellulase mixture of lignocellulose after pretreatment or the total sugar yield (hexoses and pentoses/dry weight) from both pretreatment and enzymatic hydrolysis (Table 3 and S1 File). In this study, we determined the sugar yield of 171 *Miscanthus* samples released after pretreatment with 1% NaOH or 1% H_2_SO_4_. As a result, these four *Miscanthus* species showed varied hexose yields with a CV% ranged from18.10% to 33.06%, and total sugar yields with a CV% from 11.87% to 46.05%. *M*. *sacchariflorus* had the highest average hexose yield, whereas *M*. *lutarioriparius* showed the lowest average hexose yield upon pretreatment with 1% NaOH or 1% H_2_SO_4_. Furthermore, *M*. *sinensis* exhibited the highest total sugar yield at 51% under pretreatment with 1% NaOH or 1% H_2_SO_4_ among the four species. These results suggest that diverse cell wall compositions lead to varied biomass digestibility in four *Miscanthus* species, consistent with the previous reports [14–16, 23, 28].

**Table 3:** Variation of biomass saccharification in four *Miscanthus* species.

Species	No. Accessions	Hexose released (% cellulose)				Total sugar released (% Dry matter)			
		1% NaOH	CV%^a^	1% H_2_SO_4_	CV%	1% NaOH	CV%	1% H_2_SO_4_	CV%
*M*.*sinensis*	78	51.40*	18.10	34.82	21.84	36.96	36.23	38.90	13.60
		(28.88~71.14)^#^		(22.78~59.26)		(25.42~50.83)		(24.20~50.52)	
*M*.*floridulus*	36	51.44	21.28	37.27	23.71	35.00	46.06	38.71	11.87
		(29.50~73.79)		(11.79~58.45)		(26.87~47.33)		(23.00~47.39)	
*M*.*sacchariflorus*	32	56.70	20.97	37.11	26.06	38.22	25.16	37.95	14.28
		(27.47~78.49)		(9.95~47.57)		(27.28~44.76)		(16.67~45.45)	
*M*.*lutarioriparius*	25	36.78	28.03	23.73	33.06	32.14	35.11	34.60	12.00
		(27.25~73.90)		(11.45~42.63)		(26.25~46.21)		(27.14~42.95)	
Total	171	50.27	23.55	34.15	27.46	36.08	36.89	38.06	13.65
		(27.25~78.49)		(9.95~59.26)		(25.42~50.83)		(16.67~50.52)	

* Mean value

^**#**^ Range in parenthesis.

^a^ The coefficient of variation (CV),which represents the ratio of the standard deviation to the mean, is a statistical measure of the dispersion of data points in a data series around the mean.

### Golden cutting line of *Miscanthus* distribution for high biomass saccharification

Based on the biomass enzymatic digestibility under pretreatment with 1% NaOH or 1% H_2_SO_4_, the 171 *Miscanthus* accessions were equally divided into three groups with each containing 57 accessions. We mapped the geographic locations of Group I and Group III accessions, which had relatively high (H) and low (L) biomass digestibility, respectively (Fig 2A and 2B). Surprisingly, we found that almost all *Miscanthus* accessions in Group I located in the East of China, whereas Group III distributed mainly in the West. To draw the cutting line of the geographic distribution of the Group I and Group III accessions, the probability (P) parameter for the *Miscanthus* samples with high (H) biomass digestibility in the East was calculated according to the equation: P(H|East) = P(H∩East) / P(East). The cutting line was drawn from 42°25’ N, 108°22’ E to 22°58’ N, 116°28’ E using the equation: Y = (301.46±6.26)-(2.395±0.055)X [X referring to the Longitude (E) and Y to the Latitude (N)]. Based on the cutting line with P(H|East) values at 0.800 (1% NaOH) and 0.813 (1% H_2_SO_4_), we identified that 52 *Miscanthus* accessions of Group I and 12–13 accessions of Group III were located in the east (Fig 2A and 2B). As a comparison, 91% of the *Miscanthus* accessions with high biomass saccharification distributed in the east, whereas only 21–23% of the *Miscanthus* accessions with low biomass saccharification distributed in the east. Hence, the results have demonstrated that the golden cutting line generated from the maximum P value, should be applicable for screening out of the desire *Miscanthus* accessions with high biomass saccharificaiton in the suitable growing regions.

### Typical climate factors at the locations of the *Miscanthus* accessions with high biomass saccharification

In this study, we initially collected data about 26 meteorological factors for the original locations of the *Miscanthus* accessions. Four of them, including average insolation (kWh/m2/day), average and minimum temperatures (°C), and average precipitation (mm/day) were selected for probability (P) parameter analysis of the *Miscanthus* accessions with high biomass saccharification. The 171 *Miscanthus* accessions were equally divided into three groups based on the meteorological factors of their geographical locations, i.e. Group I with high values of the meteorological factors, Group II with intermediate values, and Group III with low values. Using the cutting line of the *Miscanthus* locations described above, we calculated the P parameter for the *Miscanthus* accessions (n = 114) (Group I and Group III) exposed to high (H) average insolation in the east with P(H|East) and P(East|H) values at 0.782 and 0.754, respectively (Fig 3). These two P values indicated that the *Miscanthus* accessions located in the east of China have 78% probability of exposing to high average insolation or about 75% of the *Miscanthus* accessions exposing to high average insolation are located in the east. Furthermore, we observed that 77% (1% NaOH pretreatment) and 70% (1% H_2_SO_4_ pretreatment) of the *Miscanthus* accessions showing high biomass saccharifcaition had been historically exposed to high average insolation in the east. In addition, we calculated P(H|East) and P(East|H) values on the average and minimum temperatures and average precipitation (Additional files 3, 4 and 5). These three meteorological factors exhibited low P(East|L) and P(East|H) values ranging from 0.316 to 0.667, indicating that they did not play a major role in biomass enzymatic digestibility of the *Miscanthus* accessions. On the other hands, the *Miscanthus* accessions with the high (H) values of these three meteorological factors distributed mainly in the South of China, whereas the accessions at low (L) values distributed in the North. Therefore, the average insolation could be the unique factor that positively affects biomass enzymatic saccharification in the *Miscanthus* accessions.

### Geographical location and cell wall composition of the *Miscanthus* accessions

Since biomass saccharification is fundamentally affected by plant cell wall composition and wall polymer features in *Miscanthus*, we intended to determine the relationship between geographic distribution and cell wall composition in the *Miscanthus* accessions. As described above, 171 *Miscanthus* accessions were divided into three groups based on the levels of their wall polymer (cellulose, hemicelluloses, and lignin). 69 representative *Miscanthus* accessions were selected for the analysis of cell wall polymer features (CrI, Xyl/Ara, S/G). According to the cutting line of the *Miscanthus* locations, the probability (P) parameters were calculated for the cell wall compositions of 114 *Miscanthus* accessions and wall polymer features of 46 *Miscanthus* accessions showing relatively high (H) and low (L) values in Group I and Group III (Table 4). As the levels of the two cell wall polymers (cellulose and lignin) and three major polymer features (CrI, Xyl/Ara, and S/G) have been determined as the negative factors on biomass saccahrification in *Miscanthus*, we calculated their P(East|L) values, but P(East|H) was generated for hemicellulose due to its positive effect. As a result, relatively high P values (0.684 and 0.789) were obtained for the cellulose and hemicellulose, indicating that either low (L) cellulose level or high (H) hemicellulose contents of the *Miscanthus* accessions were located mainly in the east. By comparison, a much lower P value (0.456) was observed for lignin, suggesting that lignin might not correlate with the *Miscanthus* distribution. Among the three cell wall polymer features (Table 4), cellulose CrI showed the highest P(East|L) value (0.739), whereas Xyl/Ara of hemicellulose and S/G of lignin had P values at 0.609 and 0.696, respectively. Hence, hemicellulose level and cellulose CrI should be the major factors associated with the original locations of the *Miscanthus* accessions with high biomass enzymatic digestibility.

**Table 4:** P(East|L/H) values for cell wall compositions and wall polymer features of *Miscanthus* accessions located in the east of China.

Cell wall composition (% Dry matter)		Wall polymer features	
**Cellulose**	P(East|**L**) = 0.684	**CrI**	P(East|**L**) = 0.739
**Hemicelluloses**	P(East|**H**) = 0.789	**Xyl/Ara**	P(East|**L**) = 0.609
**Lignin**	P(East|**L**) = 0.456	**S/G**	P(East|**L**) = 0.696

*Miscanthus* biomass is increasingly regarded as one of leading biofuel feedstocks. Genetic improvement of the *Miscanthus* species will enhance their production and utilization as bioenergy crops. This study characterized four major *Miscanthus* species for their diverse geographical distribution, distinct cell wall compositions/wall polymer features, and biomass enzymatic saccharification. This study at first time determined the relationship of those three cell wall characteristics of the *Miscanthus* species with the geographical distribution and related climatic factors. These information will be very useful for the collection of *Miscanthus* germplasms and breeding desired *Miscanthus* varieties for biofuel production Notably, the golden cutting line (42°25’ N, 108°22’ E to 22°58’ N, 116°28’ E) of the *Miscanthus* distribution defined in this study will be helpful for selecting *Miscanthus* accessions with high biomass enzymatic digestibility in the east of China, which could be applied to make national policy for potentially developing bioenergy *Miscanthus* crops in China. Furthermore, as the average insolation is the unique positive factor on biomass saccharification among the four meteorological factors for the *Miscanthus* distribution, it should be considered as an important parameter for growing *Miscanthus* crops over the world.

Plant cell wall composition and wall polymer feature have significant effects on biomass saccharification. This study revealed that high hemicellulose content and low cellulose CrI level are major factors for enhancing biomass enzymatic digestion of *Miscanthus*. However, as hemicelluloses positively affect biomass saccharification by reducing cellulose CrI in *Miscanthus* [15], the cellulose crystallinity should be considered a primary target for genetic modification of plant cell walls in *Miscanthus*. In terms of the lignin level without distinct effect on *Miscanthus* distributions, we inferred that it is due to lignin playing important roles in plant resistance to various environmental stresses.

This study provides significant information on the germplasm accessions under four major *Miscanthus* species, including biomass enzymatic saccharification, cell wall compositions, and their correlation with geographical distribution and climatic factors (Table 5, Additional file 2). These information will be useful for the plant geneticists and breeders to develop superior bioenergy crops from *Miscanthus* species. Also, they will be helpful guidelines for policy makers to develop sustainable strategy for biofuel production in China and worldwide.

**Table 5:** The *Miscanthus* accessions with the highest biomass digestibility.

Species	Longitude (E), Latitude (N)	Average Insolation (kWh/m^2^/day)	Hexoses released (% cellulose)	Cell wall composition (% Dry matter)		
				Cellulose	Hemicelluloses	Lignin
*M*.*sinensis*						
Msi69	37.52° N, 121.46° E	4.31±1.26	71.14^a^	32.56±0.55	29.84±2.18	26.19±0.15
Msi69			59.26^b^			
*M*.*floridulus*						
Mfl26	29.01° N, 114.22° E	3.46±0.87	73.79	33.77±2.01	32.78±1.38	24.84±0.26
Mfl31	29.01° N, 121.17° E	4.00±1.25	58.45	27.42±0.30	30.59±0.53	20.89±0.59
*M*.*sacchariflorus*						
Msa24	36.58° N, 111.72° E	4.32±1.15	78.49	31.69±1.42	30.96±1.51	26.11±0.77
Msa32	36.58° N, 113.77° E	4.33±1.12	47.57	32.08±1.29	30.08±0.45	25.20±0.45
*M*.*lutarioriparius*						
Mlu26^c^	32.07° N, 118.85° E	3.88±0.85	73.90	29.86±1.47	30.76±1.31	25.10±0.11
Mlu26			42.63			

^a,b^ Under 1% NaOH and 1% H_2_SO_4_ pretreatments, respectively

^c^ Mlu26 has used by Xu N *et al*., 2012 [10].

## Conclusions

Based on the probability parameter calculation, a golden cutting-line has been generated to identify *Miscanthus* accessions located in the east of China with high biomass saccharification. It has indicated that either the averaged insolation or the hemicelluloses level and lignocelluose crystallinity are the main factors affecting biomass digestibility of *Miscanthus* accessions. This study can suggest the potential strategy for developing dedicated bioenergy *Miscanthus* crops in China and over the world.

## Supporting Information

S1 Fig The cutting-line of geographic locations of 114 *Miscanthus* accessions exploded under high (H, dark dot) and low (L, gray dot) averaged temperature (°C) in China. (TIF)

S2 Fig The cutting-line of geographic locations of 114 *Miscanthus* accessions exploded under high (H, dark dot) and low (L, gray dot) minimum temperature (°C) in China. (TIF)

S3 Fig The cutting-line of geographic locations of 114 *Miscanthus* accessions exploded under high (H, dark dot) and low (L, gray dot) averaged precipitation (mm/day) in China. (TIF)

S1 File All data for 171 natural *Miscanthus* accessions. The excel contains the numbers of 171 materials, geographic locations, cell wall compositions, biomass digestibility and climate factors.
(XLSX)

S1 Table Correlation coefficient of cell wall composition between 2009 and 2010 season in *Miscanthus* (n = 49). (PDF)

S2 Table Variation of hexose released (% cellulose) in four major *Miscanthus* species in East of China. (PDF)
