# Supplementary figures and images for: Distinct Geographical Distribution of the Miscanthus Accessions with Varied Biomass Enzymatic Saccharification
Source: PLoS One. 2016 Aug 17;11(8):e0160026. doi: 10.1371/journal.pone.0160026 (PMC4988763; doi:10.1371/journal.pone.0160026)

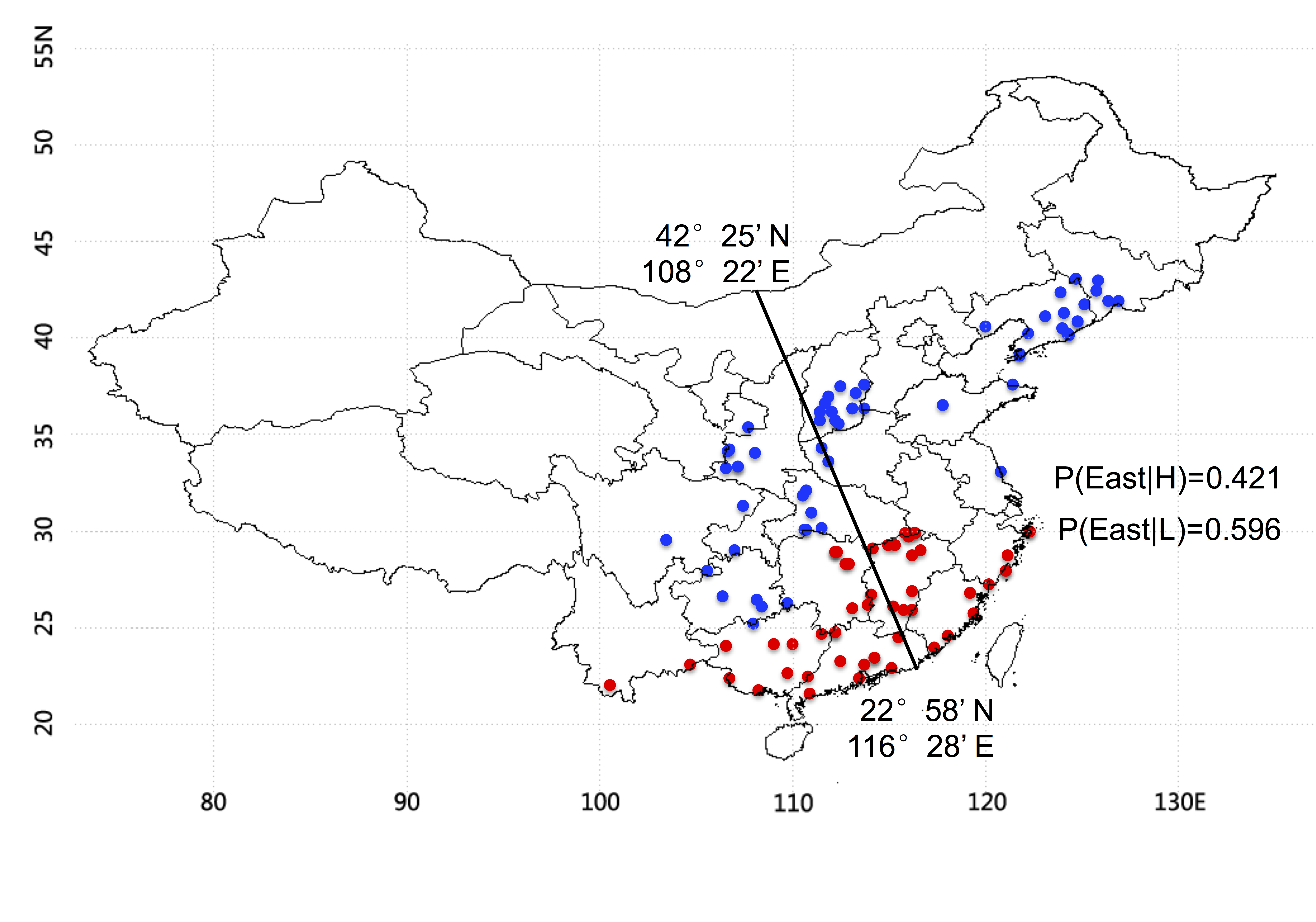

Supplement: S1 Fig — (TIF) [file pone.0160026.s001.tif]

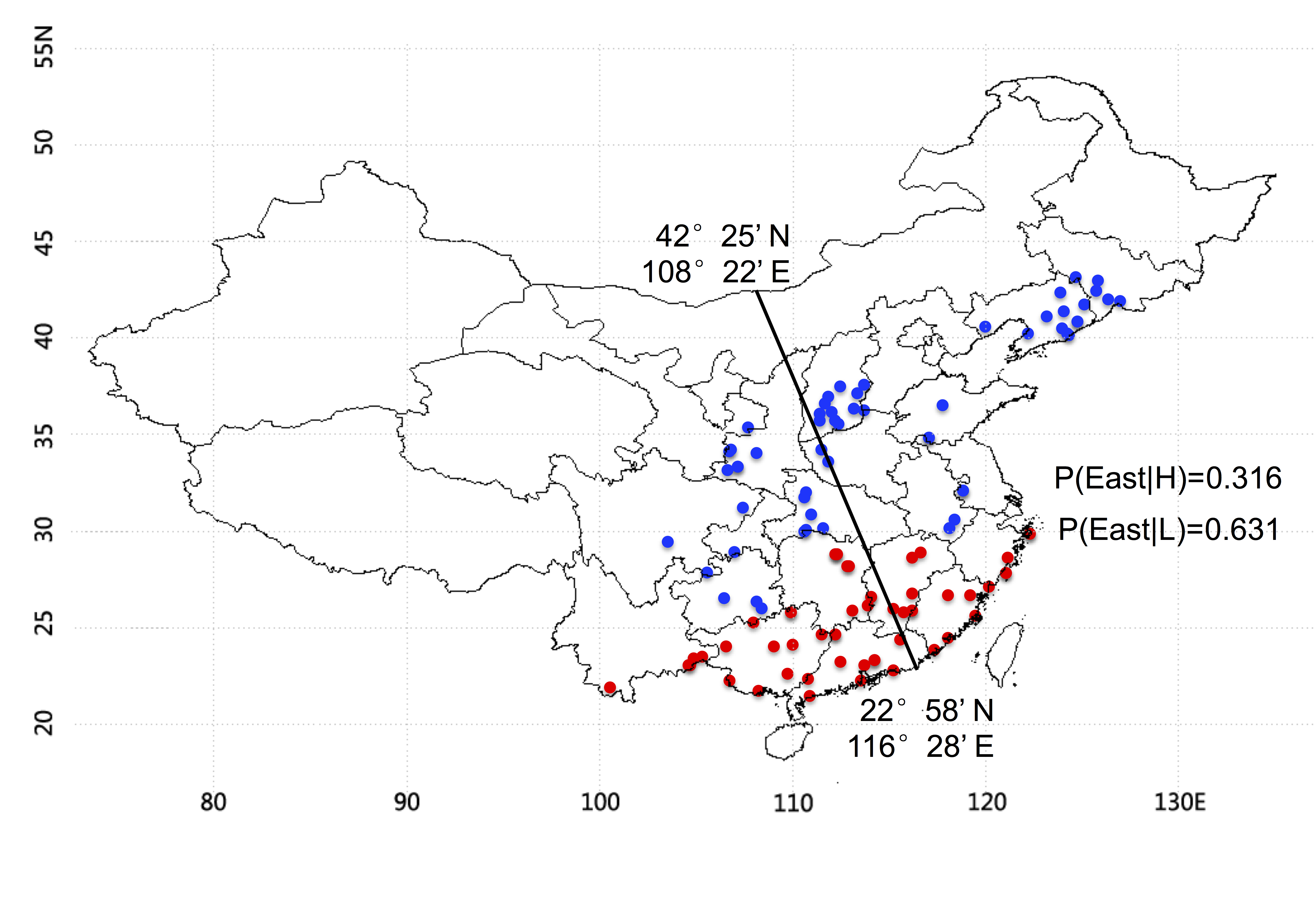

Supplement: S2 Fig — (TIF) [file pone.0160026.s002.tif]

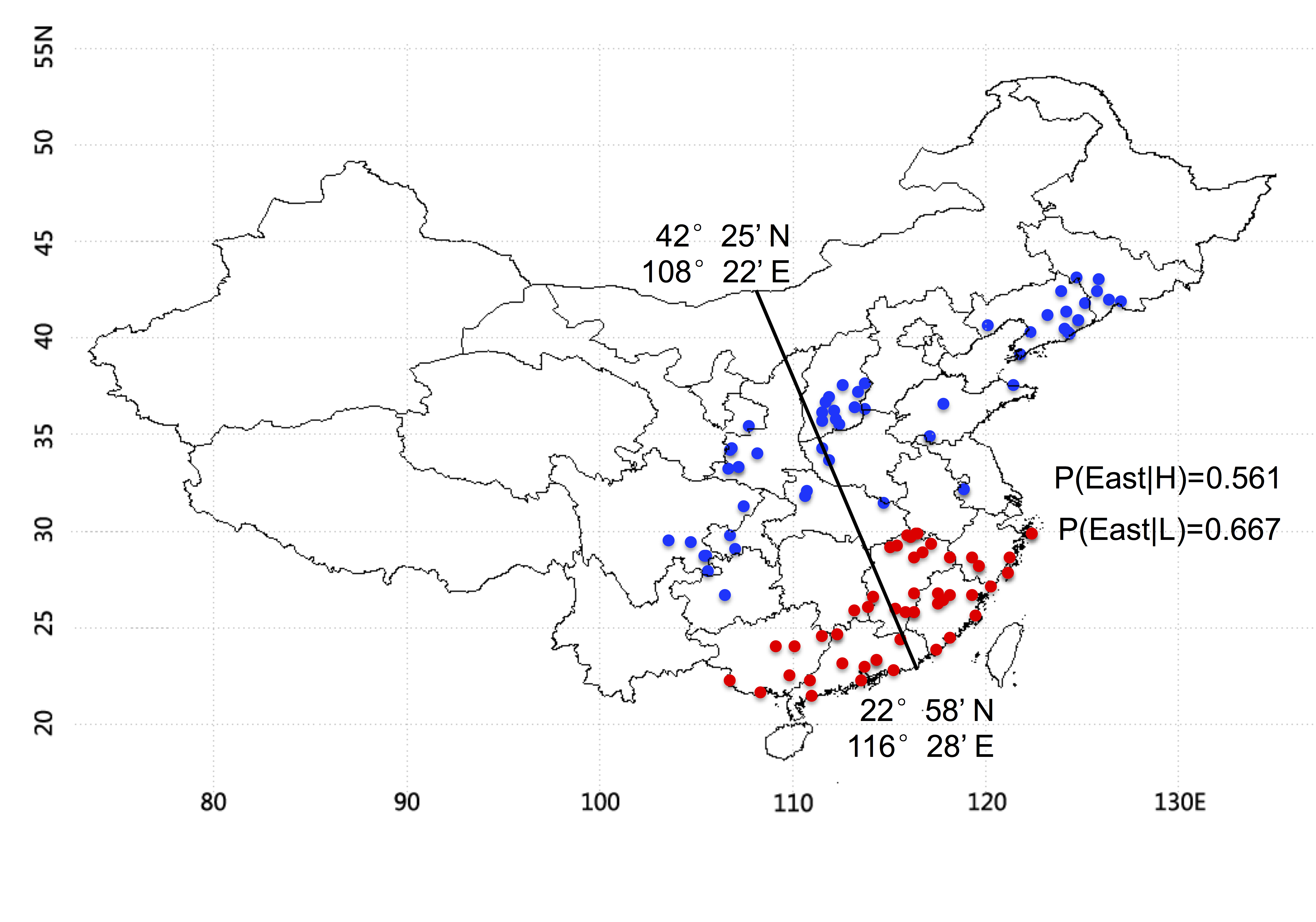

Supplement: S3 Fig — (TIF) [file pone.0160026.s003.tif]
