# Supplementary material for: Distinct Geographical Distribution of the Miscanthus Accessions with Varied Biomass Enzymatic Saccharification
Source: PLoS One. 2016 Aug 17;11(8):e0160026. doi: 10.1371/journal.pone.0160026 (PMC4988763; doi:10.1371/journal.pone.0160026)
Supplement: S1 Table — (PDF) [file pone.0160026.s005.pdf]

S1 Table.

		2009 Season		
		**Cellulose**	**Hemicelluloses**	**Lignin**
	**Cellulose**	0.521**		
**2010 Season**	**Hemicelluloses**		0.491**	
	**Lignin**			0.724**

** Significant test at $p < 0.01$.
