# Supplementary material for: Distinct Geographical Distribution of the Miscanthus Accessions with Varied Biomass Enzymatic Saccharification
Source: PLoS One. 2016 Aug 17;11(8):e0160026. doi: 10.1371/journal.pone.0160026 (PMC4988763; doi:10.1371/journal.pone.0160026)
Supplement: S2 Table — (PDF) [file pone.0160026.s006.pdf]

S2 Table.

Species	Number	1% NaOH	CV%	1% H_2SO_4	CV%
M.sinensis	36	57.97* (46.93~71.14)#	11.81	39.95 (23.44~59.26)	18.37
M.floridulus	18	58.80 (49.34~73.79)	12.77	43.20 (35.51~58.45)	15.07
M.sacchariflorus	26	60.97 (41.81~78.49)	12.63	40.62 (31.28~47.57)	10.87
M.lutarioriparius	15	40.86 (28.79~73.90)	27.38	27.53 (16.70~42.63)	27.35
Total	95	50.27 (28.79~78.49)	18.51	38.79 (16.70~59.26)	21.06

* Mean value, # Minimum and maximum values
